# The roles of cancer stem cells and therapeutic implications in melanoma

**DOI:** 10.3389/fimmu.2024.1486680

**Published:** 2024-11-14

**Authors:** Xiaoli Mu, Yixin Zhou, Yongxin Yu, Mingyi Zhang, Jiyan Liu

**Affiliations:** ^1^ The Department of Biotherapy, Cancer Center, West China Hospital, Sichuan University, Chengdu, Sichuan, China; ^2^ The Department of Plastic and Reconstructive Surgery, West China Second University Hospital, Sichuan University, Chengdu, Sichuan, China

**Keywords:** melanoma stem cells, surface marker, signaling pathway, tumor microenvironment, therapeutic advances

## Abstract

Melanoma is a highly malignant skin tumor characterized by high metastasis and poor prognosis. Recent studies have highlighted the pivotal role of melanoma stem cells (MSCs)—a subpopulation of cancer stem cells (CSCs)—in driving tumor growth, metastasis, therapeutic resistance, and recurrence. Similar to CSCs in other cancers, MSCs possess unique characteristics, including specific surface markers, dysregulated signaling pathways, and the ability to thrive within complex tumor microenvironment (TME). This review explored the current landscape of MSC research, discussing the identification of MSC-specific surface markers, the role of key signaling pathways such as Wnt/β-catenin, Notch, and Hedgehog (Hh), and how interactions within the TME, including hypoxia and immune cells, contribute to MSC-mediated drug resistance and metastatic behavior. Furthermore, we also investigated the latest therapeutic strategies targeting MSCs, such as small-molecule inhibitors, immune-based approaches, and novel vaccine developments, with an emphasis on their potential to overcome melanoma progression and improve clinical outcomes. This review aims to provide valuable insights into the complex roles of MSCs in melanoma biology and offers perspectives for future research and therapeutic advances against this challenging disease.

## Introduction

Melanoma, recognized as the most formidable skin malignancy, with an alarming global trend characterized by escalating incidence and mortality rates (1). According to International Agency for Research on Cancer (IARC), there are approximately 325,000 new cases associated with cutaneous melanoma in 2020 (2), accounting for 1.7% of all cancer diagnoses, with a potential 57,000 deaths in the same period (1). The armamentarium of melanoma treatment modalities encompasses surgical intervention, radiation therapy, chemotherapy, immunotherapy, and targeted therapy (3, 4). Due to its great heterogeneity and high metastatic potential, melanoma demonstrates a considerably restricted responsiveness to currently available therapeutic interventions (5).

CSCs represent a limited subset of neoplastic cells residing within tumor microenvironment (TME), distinguished by their intrinsic potential for incessant self-renewal, unrestricted proliferative expansion, and pluripotent aptitude for differentiation across diverse cellular lineages (6). They are also recognized as the origin of tumor relapse and the facilitators of metastatic progression, primarily due to their comparatively quiescent state and their capability to efflux chemotherapeutic agents from intracellular compartments (7, 8). Similar to other stem cells, melanoma spheroid cells, demonstrate the capacity for proliferation, differentiation, and self-renewal (9, 10). Furthermore, their substantial tumorigenic potential was corroborated through experiments involving SCID/NOD mice.

The absence of established markers for distinguishing melanoma stemness underscores the central and contentious issue in the current discourse: the identification of melanoma stem cells (MSCs). Presently, the most extensively investigated markers include CD20 (11, 12), CD133 (13–15), CD271 (15, 16), ABCB5 (17, 18), and SOX10 (19, 20); nonetheless, none of these markers has definitively demonstrated exclusive specificity for MSCs. Efforts to target these markers have led to the development of novel therapeutic strategies aimed at isolating and eliminating these resilient cells.

In addition to surface markers, targeting aberrant signaling pathways in MSCs has emerged as another promising strategy. Abnormal activation of pathways such as Wnt, Hedgehog(Hh), and Notch is commonly associated with the maintenance of MSC characteristics (21), including their self-renewal and drug resistance (22). Therapeutic interventions targeting these pathways—such as small molecule inhibitors or pathway-specific antibodies—have shown potential in disrupting the stemness and survival of MSCs, thereby curbing melanoma progression.

Moreover, recent advances in immunotherapy have further expanded the arsenal against melanoma, with a growing focus on targeting CSCs to mitigate tumor relapse and metastasis. Immune checkpoint inhibitors(ICIs), such as anti-PD-1/PD-L1 and anti-CTLA-4, have been pivotal in reactivating the immune system’s ability to recognize and attack CSCs, thereby improving clinical outcomes for many patients (23, 24). Emerging approaches like CAR-T cell therapy and CSC-targeted vaccines, seem to be a potentially powerful choice for prevention or treatment of cancers (25). These innovations not only deepen our understanding of the interplay between CSCs and the immune system but also open new avenues for developing therapies aimed at reducing recurrence and overcoming resistance in melanoma treatment.

In this comprehensive review, we synthesized recent research on cell surface markers and signaling pathways implicated in melanoma stemness, while also elucidating how MSCs interacted with the complexities of the TME. Additionally, we discussed the therapeutic implications of targeting these aspects, offering insights into potential directions for improving melanoma treatment strategies. These insights were expected to pave the way for more effective therapeutic approaches, warranting further in-depth research and exploration.

## Methods

### Literature search strategy

A comprehensive literature search was conducted to gather relevant studies for this review. The search was performed across major scientific databases including PubMed and Web of Science.

### Keywords

The search included combinations of keywords such as “cancer stem cells”, “melanoma”, “tumor microenvironment”, “biomarkers”, “signaling pathway”, “sphingolipids”, “S1P”, “exosomes”, “immunotherapy”, “Targeted therapy”, “drug resistance”, “immune modulation”, “immune cells” and “metabolic reprogramming”. Boolean operators (AND, OR) were used to refine the search, ensuring thorough coverage of all relevant topics.

### Inclusion and exclusion criteria

Studies included in this review were selected based on relevance to the impact of the TME on MSCs, interactions between immune cells and MSCs, and molecular mechanisms within the TME that influence CSC survival and function. Only peer-reviewed articles written in English were considered. Studies were excluded if they focused on non-melanoma cancers or lacked sufficient data on the interaction between the TME and MSCs.

### Time frame

The review primarily includes studies published within the past 20 years (2004-2024), ensuring a focus on recent advancements while also capturing significant earlier research that has contributed to the understanding of TME-CSC interactions.

## Identifying Biomarkers for MSCs

CSCs are believed to manifest cellular surface and intracellular indicators traditionally correlated with tissue-specific stem cells, playing a pivotal role in the generation of tumor heterogeneity (26). From a molecular point of view, MSCs can be isolated and identified by the expression of stemness-associated markers, such as surface markers, ATP-binding cassette (ABC) transporters, embryonic stem cells and intracellular markers (27). Conversely, the presence of distinct biomarkers for MSCs remains a subject of debate, primarily due to the remarkable plasticity of this malignancy and the potential coexistence of various mechanisms contributing to melanoma progression (28). As mentioned previously, MSCs can be characterized based on the expression of markers such as CD20, CD133, and CD271 et al., and the main MSCs markers proposed and studied in the previous literature are summarized as follows (**Table 1**).

**Table 1:** Function of common melanoma stem cells(MSCs) markers.

Biomarkers of MSCs	Function
**CD20**	CD20 represents a phenotype of MSCs associated with melanoma drug resistance. CD20+ cells comprise approximately 2% of the total melanoma cell population. This CD20-expressing melanoma subpopulation is distinguished by its capacity for self-renewal, differentiation into multiple cell lines, and heightened tumorigenicity.
**CD133**	CD133, a member of the pentameric transmembrane glycoproteins, is considered the most important surface marker for the recognition of mesenchymal stem cells. CD133+ cells exhibit stem cell-like properties in a variety of tumors including glioma, colon cancer, pancreatic cancer, and liver cancer.
**CD271**	CD271 is a low-affinity nerve growth factor receptor that plays an important role in promoting melanoma cell invasion and migration *in vitro*. CD271 promotes the conversion of melanoma cells from a highly proliferative and less aggressive to a less proliferative and more aggressive phenotype.
**ABCB5**	ABCB5 is a plasma membrane protein and a member of the human P-glycoprotein family. It is highly overexpressed by CSCs in various human malignancies.ABCB5 is associated with clinical tumor progression, chemoresistance and relapse in patients with malignant melanoma.
**ALDH**	ALDHs are a superfamily of detoxifying enzymes. Involved in oxidative stress response, contributing to drug resistance in melanoma. ALDH1 has been linked to the regulation of signaling pathways involved in CSC maintenance, such as the Wnt/β-catenin, Notch, and Hedgehog pathways.
**SOX10**	SOX10 is a thread-specific transcription factor that promotes the development of neural crest cells and contributes to the growth of melanoma cells.

Bolded values indicate the names of melanoma stem cell (MSC) markers, highlighting key identifiers for MSC functions within the table.

### CD20

CD20, a non-glycosylated phosphoprotein, plays a regulatory role in the proliferation and differentiation of B lymphocytes. Given its ubiquitous expression on the surface of B lymphocytes and its tendency to bind readily to antibodies without shedding, monoclonal antibodies targeting CD20 have emerged as the standard treatment for non-Hodgkin’s lymphoma. In 2005, a study found that the tumor stem cell population was enriched in the CD20(+) fraction of melanoma cells. Fang et al. (11) identified the presence of multipotent spheroid cells within melanoma cell lines. Their study has revealed that the stem cell population within melanoma cells is notably enriched in the CD20(+) fraction, thus designating it as a MSCs population. Notably, the CD20(+) fraction demonstrated a tendency to form larger spheres in comparison to the CD20(-) fraction, along with a heightened potential for mesenchymal differentiation (11). It is crucial to highlight that CD20(+) cells, which play a pivotal role in tumor stemness, constitute a relatively small fraction, representing approximately 2% of the total melanoma cell population. Schlaak et al. treated patients with metastatic melanoma by injecting the therapeutic anti-CD20 antibody rituximab at the lesion site and showed that the use of rituximab yielded excellent efficacy, accompanied by a reduction in the melanoma serum marker S-100 to levels within the physiological range (29).

### CD133

CD133, or Prominin-1, is considered to be the most important surface marker for identifying mesenchymal stem cells (30–32). Indeed, in various tumor types, such as glioma, colon cancer, pancreatic cancer, and liver cancer, CD133(+) cells consistently display characteristics resembling those of stem cells (32), underscoring the potential significance of CD133 as a marker for tumor stem cells. In the context of melanoma, only the CD133(+) subpopulation, not the CD133(-) counterpart of melanoma cells, were able to reform a Mart-1 (a characteristic melocytic marker) positive tumour in NOD-SCID mice (33). CD133 expression levels demonstrate an elevation in both primary and metastatic melanomas when contrasted with their normal pigmented nevi counterparts (34). Additionally, research findings from other studies have also indicated a correlation between increased CD133 expression levels and enhanced tumorigenicity and metastatic propensity in melanoma (30). However, doubts still abound, researchers sought to assess whether markers such as CD10, CD133, nestin and CD20 could assess the prognosis of advanced melanoma, the results showed that they were unable to detect a significant correlation between nestin or CD133 expression in melanoma and patient survival or clinical outcome (35), which implies that CD133 may not exert a significant influence as a prognostic factor.

### CD271

CD271, also termed the low-affinity nerve growth factor receptor or p75NTR, is a characteristic marker of mesenchymal stem cells (36, 37). Melanocytes are formed during the differentiation of multipotential neural crest stem cells (NCSCs) under precise regulatory mechanisms. CD271 exhibits analogous expression patterns in melanocytes, melanoma cells, and NCSCs (38). Furthermore, it plays a pivotal role in governing the preservation of cellular stemness and migratory characteristics via an intricate network of interconnected genes. The tumorigenic assay performed by Bolok et al. showed that CD271 was identified as a CSC marker that can identify and prospectively isolate MSCs (37). However, CD271(+) melanoma cells lack expression of typical melanoma cell surface markers such as TYR, MART, and MAGE, leading to the speculation that CD271+ melanoma cells may be in a state of incomplete differentiation. In addition, CD271 regulates phenotypic transition, a process that results in rapid and reversible conversion of the proliferative state to an invasive state or of the non-stem cell state to a stem cell state, by mechanisms that are not entirely understood (38). Cheli et al. considered that CD271 was not a perfect marker for MSCs, their study showed that not all CD271+ cells were tumorigenic, only the slow-growing CD271+ cell subpopulation was highly tumorigenic (39).

### ABCB5

ABCB5, a member of the ABC family of transporter proteins, is highly expressed in CD133(+) melanoma cells. It is considered to be one of the markers of MSCs and is closely associated with chemotherapy resistance in MSCs (40, 41). In 2008, Schatton et al. confirmed that ABCB5 can be used as a molecular marker for MSCs, ABCB5(+) melanoma cells had a higher tumorigenic potential than the ABCB5(-) somatic cell population in subsequent mouse xenograft trials, which re-established clinical tumour heterogeneity. *In vivo* genetic genealogy tracing demonstrated the specific ability of the ABCB5(+) subpopulation to self-renew and differentiate compared to ABCB(-), as ABCB(+) cancer cells produced both ABCB5(+) and ABCB5- progeny, whereas the ABCB5(-) tumor population produced only ABCB5(-) cells (42). A study by Wang et al. confirmed that ABCB5 is a key factor in promoting melanoma metastasis. ABCB5(+) malignant MSCs showed a higher metastatic potential compared to ABCB5(-) melanoma subpopulation (43).

### ALDH

Aldehyde dehydrogenase (ALDH) is a polymorphic enzyme family responsible for the oxidation of aldehydes to carboxylic acids. ALDH has also emerged as a promising marker for CSCs, playing a role in resistance to various chemotherapeutic agents and immune responses across a range of human solid tumors (32, 44, 45). The human ALDH superfamily comprises 19 isoforms, each with distinct biological functions beyond their enzymatic role in detoxifying bioaldehydes and xenobiotics. Among these, several isoforms are closely associated with the characterization of CSCs and the acquisition of malignant properties and drug resistance. For instance, the ALDH1 family—particularly ALDH1A1, ALDH1A2, and ALDH1A3—has been identified as a critical marker for normal stem cells and CSCs. ALDH1 plays a key role in retinoic acid (RA) signaling, which is essential for maintaining the “stemness” properties of CSCs (46). The high ALDH activity detected in the Aldefluor assay is often attributed to ALDH1A1, which contributes to the aldehyde-induced fluorescence staining (47, 48). However, recent studies have shown that ALDH1A3 also significantly contributes to Aldefluor positivity, indicating a broader role for ALDH1 isoforms in CSC characterization (49). ALDH2, another isoform, has been strongly correlated with alcohol-related tumor formation, while ALDH3A1 is implicated in drug resistance through the oxidation of chemotherapeutic agents like oxazaphosphorines, including cyclophosphamide (CP) (47). In melanoma, ALDH(+) cells have been found to be more tumorigenic compared to ALDH(-) cells. Knockdown of ALDH expression using siRNA or shRNA leads to cell cycle arrest, apoptosis, reduced cell viability *in vitro*, and reduced tumorigenesis *in vivo* (50). Nonetheless, it is noteworthy that both ALDH(+) and ALDH(-) melanoma cell subpopulations displayed comparable tumorigenic capabilities in both *in vivo* and *in vitro* assays. Additionally, both subgroups exhibited similar responsiveness to anti-melanoma drugs, including dacarbazine and lexatumumab (51). Tumors from ALDH(-) cells largely maintained the parental ALDH(-) phenotype *in vivo* after 2-3 passages. This reaffirms that the observed tumorigenicity is indeed an inherent trait of ALDH(-) cells and is not attributed to inadequate segregation of the two distinct subpopulations. In contrast, tumors originating from ALDH^+^ cells display a mixed population—predominantly ALDH(+) cells, with 20-40% of cells lacking ALDH activity (51). While ALDH(+) melanoma cells show a higher capacity to generate phenotypic heterogeneity, the functional implications of this trait remain unclear. The inability of the ALDH phenotype to distinguish between cells responsible for tumor initiation and therapy resistance suggests that it may not necessarily mark the more aggressive subpopulations within malignant melanoma. These findings imply that the ability to reestablish tumor heterogeneity is not inherently tied to a more aggressive phenotype. Therefore, further research is needed to determine whether ALDH can serve as a reliable marker for MSCs.

### SOX10

SOX10 is in the high-mobility-group (HMG)-box family of transcription factors and plays an important role in the development of melanocytes and other neural crest cells (52). The researchers conducted subcutaneous injections of melanoma cells with normal SOX10 expression and melanoma cells with SOX10 silenced by shRNA in immunodeficient NOD/SCID or nude mice. After an eight-week period, the melanoma cells expressing SOX10 consistently led to the development of substantial *in vivo* tumors (11 out of 14). In contrast, none of the subcutaneously injected melanoma cells expressing SOX10 shRNA (0 out of 16) resulted in tumor formation, even after an additional six weeks of observation.This strongly indicate that the silencing of SOX10 is highly efficacious in preventing the formation of tumors in melanoma cells in an *in vivo* setting (53). SOX10 also plays a significant role in mediating resistance to BRAF inhibition in melanoma. The downregulation of SOX10 triggers the activation of TGF-β signaling, which results in the upregulation of epidermal growth factor receptor (EGFR) and platelet-derived growth factor receptor β (PDGFRB), ultimately leading to the development of a slow-cycling phenotype. This, in turn, contributes to resistance against BRAF and MEK inhibition by bestowing survival signals that are independent of the MAPK pathway (54). Willis et al. conducted a study to investigate the staining sensitivity and specificity of SOX10 as a marker for metastatic melanoma. Their research focused on sentinel lymph nodes that had been previously diagnosed as positive or negative. The results revealed that SOX10 immunostaining successfully identified metastatic melanoma in all examined cases, achieving a 100% detection rate. Additionally, a statistically significant increase in staining intensity for SOX10 was observed when compared to S100 protein, HMB-45, and Melan-A (with respective p-values of 0.000, 0.000, and 0.003) (20). The preceding research indicates that SOX10 has the potential to serve as a marker for MSCs.

## Major signaling pathways in MSCs

**Figure 1:**
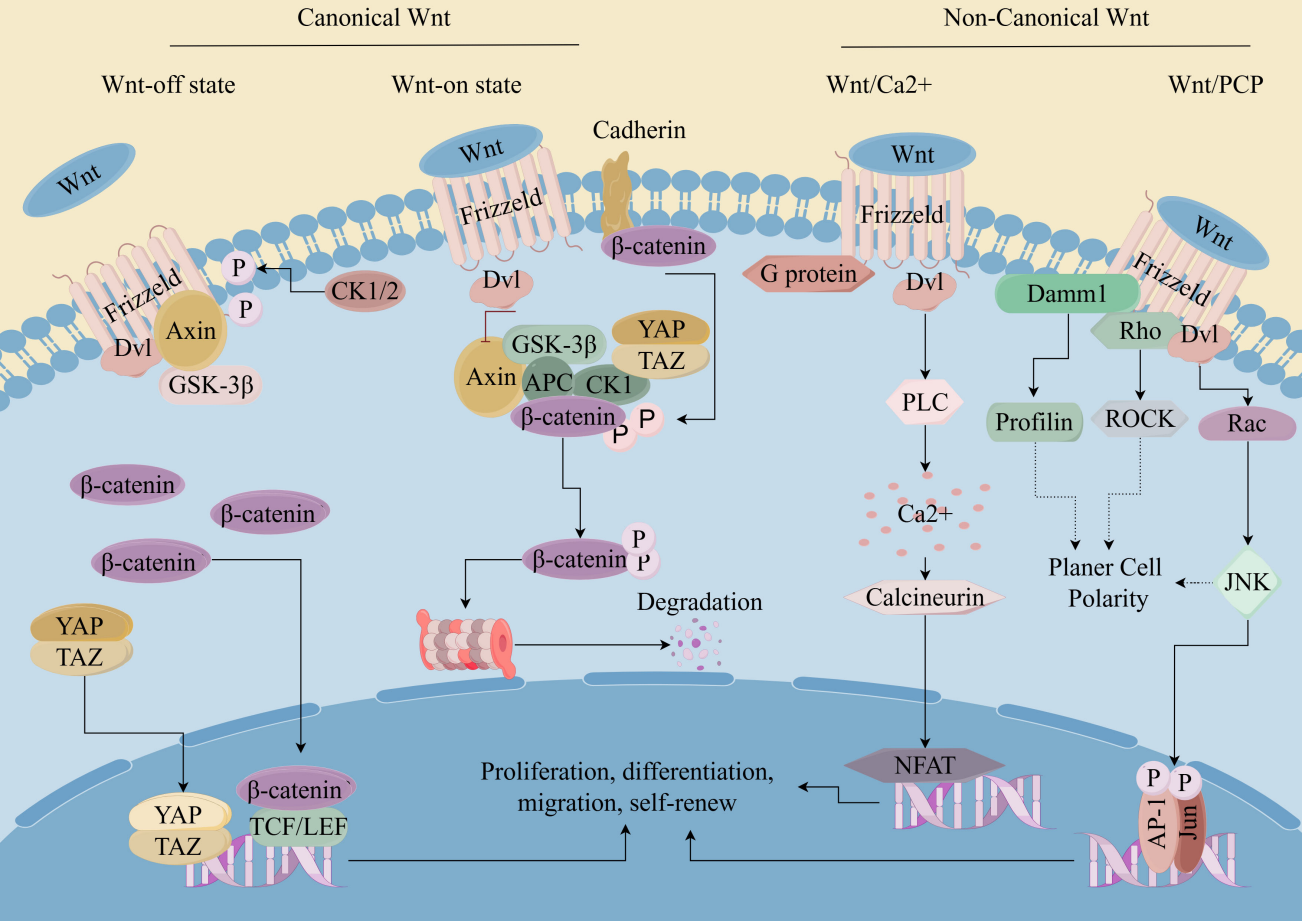
Wnt/β-catenin pathway in CSCs. (By Figdraw). Wnt ligands bind to cell surface receptors, leading to the stabilization and accumulation of β-catenin in the cytoplasm. Subsequently, β-catenin translocates into the nucleus, where it acts as a coactivator for transcription factors of the T-cell factor/lymphoid enhancer factor (TCF/LEF) family. This leads to the activation of target gene expression, which regulates cell proliferation, survival, and differentiation. Dysregulation of the Wnt/β-catenin pathway has been implicated in various diseases, including cancer, where aberrant activation of the pathway contributes to tumor initiation, progression, and metastasis.

**Figure 2:**
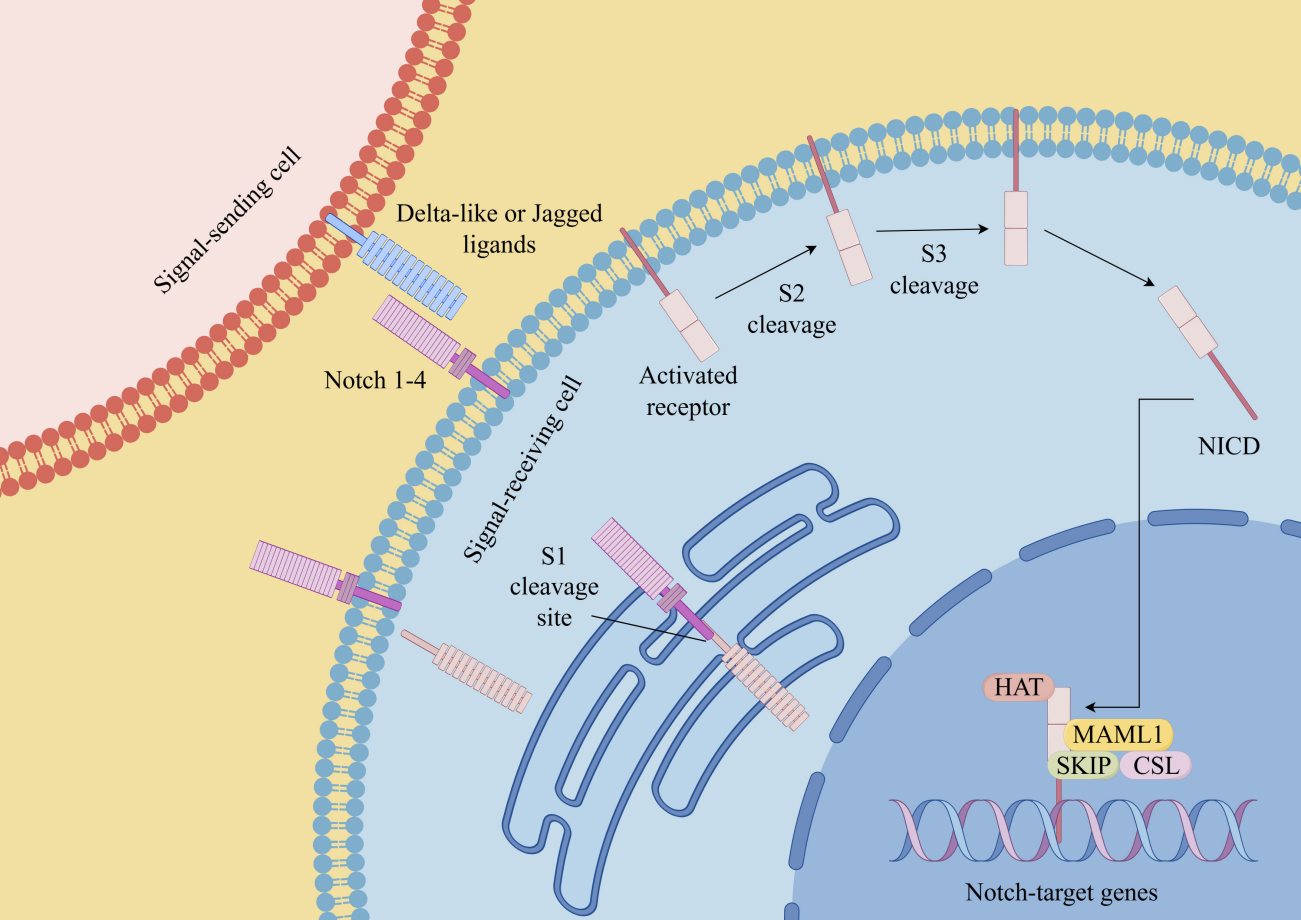
Notch signaling pathway in CSCs (By Figdraw). Notch receptors (Notch 1-4 in mammals) interact with ligands (such as Delta-like ligands and Jagged/Serrate ligands) on neighboring cells. This interaction triggers a series of proteolytic cleavage events that release the intracellular domain of Notch (NICD). NICD then translocates into the nucleus, where it forms a transcriptional activation complex with other proteins, including the CSL (CBF1/Suppressor of Hairless/Lag-1) transcription factor. This complex regulates the expression of target genes involved in various cellular processes. Dysregulation of the Notch signaling pathway has been implicated in numerous diseases, including cancer, neurodevelopmental disorders, and cardiovascular diseases.

**Figure 3:**
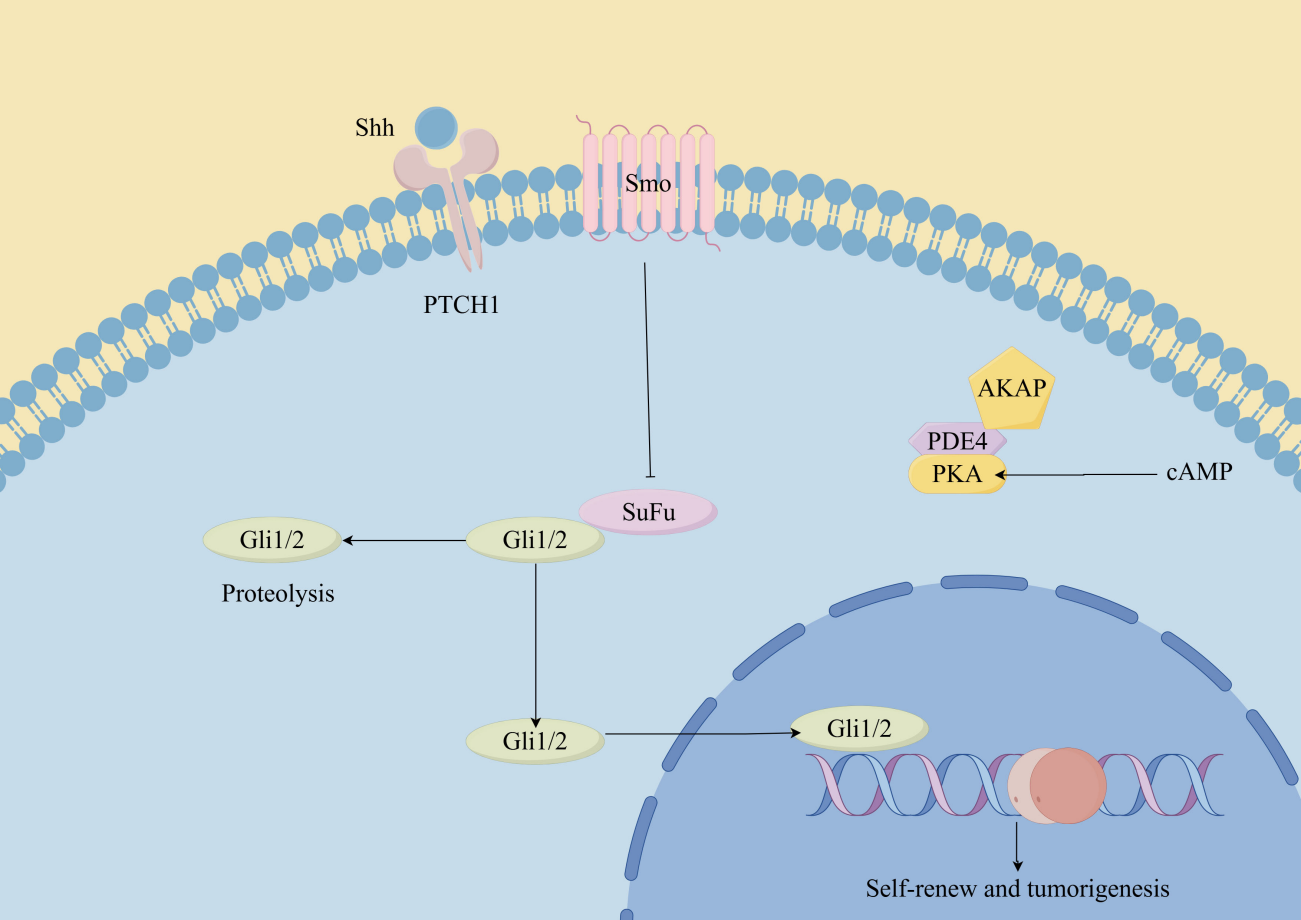
Hedgehog signaling pathway in CSCs. (By Figdraw). Hh ligands (such as Sonic Hedgehog, Indian Hedgehog, and Desert Hedgehog) bind to the Patched-1 (Ptch1) receptor, relieving its inhibition on Smoothened (Smo). This leads to the activation of downstream signaling cascades, including the Glioma-associated oncogene homolog (Gli) transcription factors. In CSCs, dysregulated Hh signaling can promote self-renewal, proliferation, survival, and resistance to therapy. Additionally, the Hh pathway has been implicated in maintaining the stemness and plasticity of CSCs, as well as promoting tumor initiation, progression, and metastasis.

Many signaling pathways critical for regulating the survival, proliferation, self-renewal, and differentiation characteristics of normal stem cells experience aberrant activation or suppression during tumorigenesis or within the realm of CSCs (55). Mutations in the driving signals of CSCs can trigger the activation of tumor growth-driving pathways, which is the most common mechanism of tumor progression and drug resistance (28). Thus far, various signaling pathways have been identified as participating in the biological functions of MSCs, including Wnt, Notch, and Hh pathways(**Figures 1**–**3**), which are succinctly delineated below.

### Wnt signaling pathways

The Wnt signaling pathway is a crucial regulatory pathway that governs various cellular functions across different biological contexts, including embryonic development, tissue homeostasis, stem cell maintenance and cell proliferation and differentiation (56). The most prominent Wnt signaling pathways include canonical pathways known as the Wnt/β-catenin pathway and non-canonical pathways such as the Wnt/PCP (planar cell polarity) pathway, the Wnt/Ca2+pathway, and the WNT/RTK (receptor tyrosine kinase) pathway (57). Both canonical and non-canonical WNT signaling cascades play fundamental roles in shaping the development and evolution of CSCs (58). Active Wnt/β-catenin signaling was reported in ∼30% of melanoma tumors, indicating a potentially specific role for this signaling pathway in this aggressive type of cancer (59). Prior research has demonstrated that curtailing the heightened activation of β-catenin, a key gene in the Wnt signaling pathway, or its inactivation by inhibiting the APC gene can reduce the accumulation of β-catenin in cells, thus inhibiting the ability of tumor stem cells to self-renew and proliferate and differentiate, and restoring the sensitivity of CSCs to radiotherapy (60–62). The expression of the Frizzled 3 receptor (FZD3) is integral to the processes of proliferation and differentiation of melanocyte derivatives in an *in vitro* setting. It was established that FZD3 is essential for melanoma oncogenesis and establishes a positive feedback mechanism for activating the MAPK signaling network by initiating the non-canonical WNT pathway. Downregulation of FZD3 diminished the growth, colony-forming potential and invasive ability of melanoma cells, besides, the inhibition of FZD3 expression suppressed melanoma initiation and growth *in vivo*. Moreover, the clinical association of FZD3 expression with melanoma progression and reduced patient survival has been observed (63).

### Notch signaling pathways

Similar to the Wnt signaling pathway, the Notch signaling pathway also assumes a significant role in the regulation of the maintenance and self-renewal of CSCs (64). The Notch pathway is composed of four different transmembrane receptors, Notch1 to Notch4, and their membrane-bound ligands, Jagged1, Jagged2, and Delta1, Delta3, and Delta4 (65). Previous studies have shown that Notch1 is highly expressed in 50-60% of melanomas and 65% of melanoma cell lines, while it is very low or undetectable in normal melanocytes and pigmented nevi (66). In a separate study, researchers have conducted an analysis of Affymetrix expression profiles employing a dataset derived from 44 samples obtained from patients diagnosed with metastatic melanoma. The findings from this analysis indicated a potential linkage between the activation of the Notch signaling pathway and the prognosis of individuals afflicted with metastatic melanoma (67, 68). Huynh et al. used RO4929097(a novel γ-secretase inhibitor) to target Notch signaling, the results revealed that it affected the oncogenic and stem cell-like properties of primary melanoma cells. Furthermore, through the utilization of *in vivo* tumorigenic assays, their research unequivocally substantiated the capability of RO4929097 to effectively curtail the growth of both primary and metastatic melanoma cells (67). This observation further underscores the potential of targeting the Notch signaling pathway as a promising therapeutic strategy for the eradication of melanoma.

### Hedgehog-GLI signaling pathways

The Hh signaling pathway is a well-conserved cascade essential for embryonic development, tissue homeostasis, and the maintenance of stem cells (69, 70). The Hh signaling pathway exhibits distinct functions in different types of cancer (71). Throughout tumor development, Hh signaling plays three major roles: driving tumor initiation, promoting tumor growth, and regulating residual cancer cells following therapy (55). Additionally, emerging evidence suggests that the Hh pathway is involved in promoting the self-renewal of CSCs (72). Evidence indicates that the Hh pathway is crucial for the oncogenic characteristics of melanoma, SHH-GLI signaling regulates the proliferation and survival of human melanoma (73). In 2012, Roberta et al. demonstrated for the first time that blocking of the Hh-GLI pathway decreased self-renewal and tumorigenicity of ALDH high MSCs (61), suggesting that the Hh-GLI1 signaling pathway significantly influences the self-renewal and tumorigenesis of MSCs. Additionally, the investigators observed that targeting the Hh transcription factors GLI1 and GLI2 restored the sensitivity of human melanoma cells resistant to vemurafenib and even inhibited acquired chemotherapy resistance in melanoma patients (74).

### Interaction between MSCs and tumor microenvironment

Increasing evidence indicates that the TME critically regulates the maintenance of CSCs, thereby controlling cancer progression and metastasis (75). The TME is a markedly heterogeneous and intricate milieu, comprising a complex network of cells and extracellular macromolecules. This encompasses stromal cells, immune cells, epithelial cells(ECs), and supporting cells within the extracellular matrix (ECM) (76). Moreover, various microenvironmental factors, such as the perivascular niche and hypoxia, have also been substantiated (77). The constituents within the TME play a pivotal role in facilitating the growth, sustenance, and differentiation of CSCs (78). Furthermore, they are involved in conferring therapeutic resistance by shielding tumor cells from damage caused by treatment interventions (79, 80). It has also been shown that CSCs also contribute to the re-establishment of the TME by transdifferentiating into a spectrum of normal stroma-like cells, such as vascular ECs, vascular pericytes or fibroblasts (81, 82). Understanding the intricate interactions within the TME, including those between CSCs and various cellular components, is crucial for unraveling the mechanisms that underlie tumor progression and therapeutic resistance.

### Immune cell interactions

MSCs interact with immune cells in the TME to evade immune detection and promote tumor growth.

#### T Cells

Cytotoxic T Cells (CD8+ T cells) play a central role in targeting and killing melanoma cells, including MSCs (83). However, MSCs can evade immune surveillance by downregulating MHC molecules and upregulating immune checkpoint molecules such as PD-L1 and CTLA-4, which suppresses the activity of cytotoxic T lymphocytes (CTLs). For example, CSCs in mouse B16 melanoma models have been shown to express higher levels of PD-L1 compared to their non-CSC counterparts (84). CTLA-4, a key immune checkpoint, acts during the early stages of T cell activation and serves as a crucial negative regulator of T cell-mediated immune responses. In melanoma cell lines B16-F0 and B16-F1, approximately 30-40% of CSCs expressed CTLA-4, and blockade of CTLA-4 suppressed stem cell-like properties and significantly inhibited spheroid formation *in vitro* (85). Additionally, ABCB5+ melanoma cells have been reported to exhibit markedly reduced or absent expression of MHC class I molecules, allowing these cells to escape recognition by CD8+ T cells and thereby impairing the anti-tumor immune response (86). It’s worth mentioning that CSCs’ own oncogenic signaling pathways may also contribute to immune escape. For instance, activation of the WNT/β-catenin signaling pathway in melanoma correlates with deletion of the T-cell gene expression signature (87, 88). Additionally, MSCs secrete immunosuppressive cytokines such as TGF-β and IL-10, which promote the recruitment and expansion of Regulatory T cells (Tregs) and Myeloid-derived suppressor cells(MDSCs), and reduce their efficiency in attacking tumors (89–91).

#### Tregs

Tregs suppress anti-tumor immune responses, creating an immunosuppressive environment that supports MSCs survival and maintenance. They are often recruited by MSCs through cytokines like TGF-β and IL-10 (83).

#### Tumor-associated macrophages

In melanoma, TAMs represent a predominant immune cell population within TME and play a critical role in carcinogenesis, metastasis, and drug resistance (92, 93). TAMs contribute to these processes by promoting angiogenesis, facilitating tumor cell invasion and metastasis, inducing immunosuppression, and enhancing resistance to chemotherapy through the secretion of various cytokines and remodeling of the ECM (94, 95). Moreover, MSCs also promote macrophages conversion to the tumor-supportive M2 phenotype, and impair dendritic cell function, reducing antigen presentation and T cell activation (96).

#### Myeloid-derived suppressor cells

MDSCs are potent suppressors of T cell responses and are known to create an immunosuppressive niche that protects MSCs. In addition, MDSCs are critical in driving epithelial-mesenchymal transition (EMT) and enhancing stemness of CSCs (97). The accumulation of MDSCs in peripheral blood and within the TME has been closely associated with disease progression, diminished T cell activity, and poorer prognosis in melanoma patients (83, 98).

#### Dendritic cells

While DCs are essential for antigen presentation and initiating anti-tumor immune responses, CSCs can impair DC function, reducing their ability to activate T cells. CSCs can release cytokines like IL-10, which hinder DC maturation and promote an immune-tolerant environment (97, 99).

#### Natural killer cell

NK cells are key effector lymphocytes in the innate immune system and play a crucial role in protecting the host from tumor invasion (100). They interact directly with tumor cells and CSCs or indirectly with other cells to regulate tumor growth within the TME (101). NK cells are capable of targeting MSCs due to their ability to recognize stressed cells that lack MHC class I expression. However, MSCs can evade NK cell activity by releasing immunosuppressive factors that inhibit NK cell function. For instance, Integrin-like protein 1 (ITGBL1), a factor highly expressed in MSCs, has been found to impair NK cell cytotoxicity and promote melanoma progression (102).

#### Neutrophils

Neutrophils can exhibit both pro-tumor (N2) and anti-tumor (N1) activities. In the case of MSCs, they often adopt an immunosuppressive phenotype (N2), which supports tumor progression. Soluble factors like TGF-β, IL-6, and IL-8, secreted by MSCs, facilitate neutrophil recruitment and induce a shift toward an N2 phenotype. This process is driven by the activation of ERK, p38, and STAT3 signaling pathways, alongside the upregulation of CXCR2 and NF-κB (101). In addition, studies have shown that in melanoma patients, the more neutrophils there are, the higher the risk of progression and death (103).

### Extracellular matrix and stromal cells

#### Cancer-associated fibroblasts

Within the TME, fibroblasts become activated in response to various inflammatory cytokines produced by cancer cells, host immune cells, and stromal cells. These activated fibroblasts are commonly referred to as CAFs (104). CAFs secrete growth factors, foster angiogenesis, remodel the ECM, facilitate metastasis, and modulate immune infiltration, creating a supportive niche for tumor cells (105, 106). Melanoma-associated fibroblasts reduce melanoma cells’ sensitivity to NK-mediated lysis by secreting active MMPs (107). Apart from these, they also influence key pathways like Notch and Wnt, maintaining CSC stemness and differentiation. In 2018, Su and colleagues defined and isolated a functional subpopulation of CAFs using specific cell surface markers, namely CD10 and GPR77. Their findings suggested that targeting specific subpopulations of CAFs could represent an effective therapeutic strategy for addressing solid tumors driven by CSCs (108).

#### Extracellular matrix

The ECM serves as the principal structural component within the TME, forming a network of biochemically distinct elements such as fibronectin, glycoproteins, proteoglycans, and polysaccharides (109). It governs melanoma phenotypic transitions, metastatic processes, and therapeutic resistance through direct influence on intracellular signaling pathways like integrin, Notch, Wnt/β-catenin, TGF-β, and Hippo/YAP pathways (110). The ECM also affects immune cell recruitment, allowing CSCs to evade immune surveillance. The dense ECM acts as a physical barrier, preventing CTLs and NK cells from infiltrating the tumor core. Additionally, ECM components and CAFs secrete chemokines that attract Tregs and MDSCs, while also sequestering chemokines to hinder effector immune cell recruitment (111).

### Soluble factors and metabolites

Melanoma cells can shape their microenvironment through both direct cellular interactions and the release of soluble factors, which are essential for their growth and metastatic potential (112). These soluble factors include cytokines, chemokines, growth and angiogenic factors and other signaling molecules (113). For instance, factors released by macrophages can stimulate nearby melanoma cells, leading to an increased production of melanoma inhibitory activity (MIA) *in vitro*. This elevated MIA, in turn, enhances the invasive capacity of melanoma cells by modulating their attachment to the ECM (114). Additionally, TGF-β, IL-6, and IL-8 released by CAFs and TAM can promote CSCs self-renewal and tumor progression by activating pathways like STAT3, NF-κB, and Wnt (80, 81). VEGF and other angiogenic factors secreted by TME cells enhance neovascularization, creating a vascular niche that further supports CSCs survival and expansion (115). Conversely, MSCs can contribute to an inflammatory microenvironment within the TME by secreting cytokines and chemokines, such as IL-6, IL-8, and TNF-α. These inflammatory factors recruit immune cells and hematopoietic stem cells into the TME, fostering an environment conducive to tumor growth and metastasis (112). Moreover, these soluble factors not only promote CSCs growth but also contribute to the establishment of an immunosuppressive microenvironment by recruiting Tregs and MDSCs, thereby inhibiting effective immune surveillance (83).

#### Metabolic adaptations

The metabolic adaptations of MSCs are crucial for their survival within the nutrient- and oxygen-limited conditions of the TME. These adaptations include shifts in glycolysis, oxidative phosphorylation, and lipid metabolism, enabling MSCs to efficiently manage energy production and survive under stress. Metabolic reprogramming of tumor cells due to genetic mutations is a key factor in the formation of TME, which is characterized by the “Warburg effect”, a predominantly aerobic glycolytic mode of energy supply (116). These metabolic changes can enhance MSCs survival and their ability to resist harsh conditions in the TME (117). In addition to increased glucose uptake and aerobic glycolysis, tumor cells require lipid metabolic reprogramming to enhance their biological behavior. Lipids are a source of energy and the structural basis of all membranes. However, lipids have also emerged as mediators that not only influence classical oncogenic signaling pathways but also contribute to the development of melanoma (118). Moreover, sphingolipids, particularly sphingosine-1-phosphate (S1P), are bioactive lipids that play a critical role in the regulation of MSCs and their interactions with the TME. S1P promotes Stat3- and Akt-mediated tumor cell growth while upregulating Bcl-2/Bcl-xL, resisting p53-mediated apoptosis, and stimulating a vicious cycle of tumorigenesis (119). In melanoma, sphingolipid signaling can enhance MSCs survival by promoting resistance to apoptosis and facilitating interactions with other cells in the TME (120).

### Extracellular vesicles

#### Tumor-derived EVs

Melanoma cells produce various EVs, including exosomes (EXOs), microvesicles and apoptotic bodies (121). These EVs are carriers of proteins, lipids, and nucleic acids, such as mRNAs, microRNAs (miRNAs), and long non-coding RNAs (lncRNAs), which contribute to pro-tumor processes, including angiogenesis, immune modulation, and alteration of tissue microenvironments (122). For instance, MSCs can enhance the metastatic colonization of non-MSCs through exosomal transfer mechanisms, thereby improving the transfer efficiency (123). Additionally, another study showed that Melanoma-Derived Exosomes (MEXs) also induced vascular leakage at pre-metastatic sites and reprogrammed biofilm progenitor cells to a pro-angiogenic phenotype (124). Studies have shown that MEXs played an important role in regulating immune responses, evading the immune system and suppressing multiple immune system functions by either directly impairing the function of immune effector cells or indirectly stimulating regulatory cells (125–127). In addition, tumor microvesicles (TMVs) also play multiple roles in disease development and dissemination, such as transferring growth factor receptors, enhancing cell viability, inducing angiogenesis, evading immune detection, and generating drug resistance (128). Exosomes released from tumor cells and stromal cells effectively promote CSCs to remain stem and tumorigenic (129): some tumor-derived exosomes are able to carry stem cell-related genes, such as OCT-4, SOX-2, NOTCH1, and NANOG, or promote their expression by mediating lncRNA/microRNA to enhance the stem cellularity of CSCs and maintain tumor heterogeneity (130).

#### TME-derived Evs

EVs released by various cells within the TME can influence the phenotype of tumor cells, affecting their growth, aggressiveness, metastatic potential, and responsiveness to therapy (131). For instance, cells like TAMs and CAFs secrete exosomes that deliver signaling molecules to MSCs, affecting their proliferation, invasiveness, and immune evasion capabilities (132). Exosomes derived from CAFs have been shown to alter cancer cell metabolism, shifting it away from oxidative phosphorylation towards glycolysis and glutamine-dependent reductive carboxylation, which supports tumor growth under conditions of nutrient stress (130, 133). In addition, the direct cytotoxic and cytostatic effects of immune cell-derived EVs on melanoma have been confirmed through both *in vitro* and *in vivo* studies (130). Zhu et al. showed that NK cell-derived exosomes have a cytotoxic effect on melanoma cells and delay tumor progression (134).

### Neurons and nerve fibers

Neurons and nerve fibers are critical components of the TME, playing significant roles in immunomodulation and the regulation of various signaling pathways in tumor cells and other cellular elements within the TME (135). These neural elements are key facilitators of pathways that drive tumor growth and metastasis (136). The interactions between neurons, nerve fibers, and the TME can be broadly categorized into two main types: (1)Perineural Invasion (PNI), which describes the process where tumor cells invade and grow along nerves. PNI is a common feature in many solid tumors and is associated with a poor prognosis; and (2) Neural-Tumor Interactions, which refer to the biochemical communication among immune cells, malignant cells, and nerve fibers within the TME (135, 137). Moreover, nerve fibers within the TME can secrete a variety of growth factors, neurotrophins, matrix metalloproteinases, neuropeptides, and neurotransmitters, which activate membrane receptors on tumor cells and support tumor progression (138). Recent studies have highlighted the role of the sympathetic nervous system (SNS) in melanoma progression through its action on β-adrenergic receptors (β-ARs) expressed on tumor cells (135). Activation of these receptors triggers the release of pro-tumorigenic cytokines and metalloproteinases in melanoma cell lines, which further facilitates melanoma growth and metastasis (139).

### Endothelial cells

Endothelial cells (ECs) play a critical role not only in vascular functions such as alloimmunity, immune cell recruitment, immune tolerance, and vascular inflammation, but also in modulating immune responses across various tissues and organs (140). In malignant tumors, angiogenesis is primarily driven by multiple pro-angiogenic factors, including growth factors, cytokines, and ECM proteins, which act on vascular ECs (141). In melanoma, increased ALDH1A1 expression and activity upregulates the release of pro-angiogenic factors, and these factors modulate the angiogenic profile of ECs by rearranging the Notch pathway (142). Compared to non-tumor ECs, those within the tumor environment exhibit enhanced proliferation, migration, and tube formation in response to these stimuli. Furthermore, they show increased resistance to therapy under the influence of VEGFA signaling (101, 143). Additionally, ECs derived from CSCs have been identified in various solid tumors, such as gliomas, renal cancers, and breast cancers (81, 144, 145). CSCs can differentiate not only from epithelial cells but also from ECs (145). This further supports the hypothesis that the CSC population has stem cell characteristics associated with tumor growth and vascularization.

#### Hypoxia

Hypoxia-induced CSC formation is a mechanism for tumor therapy resistance and progression (146). Hypoxia-inducible factors (HIFs) not only mediate the transcriptional response of normal and tumor tissues to local hypoxia, but also promote tumor development by altering cellular metabolism and stimulating angiogenesis. HIF-1α, in particular, orchestrates responses to hypoxia, including the upregulation of the melanocyte-specific transcription factor MITF, promoting melanoma growth (147). Hypoxia-induced HIFs also promote EMT, a process crucial for the acquisition of invasive properties in MSCs. HIFs directly induce the expression of EMT-related transcription factors like TWIST, SNAIL, and ZEB1, which facilitate the transition to a more mesenchymal phenotype, increasing migratory and invasive capabilities. Ghosh et al. showed that under hypoxic conditions, TAMs and Tregs achieve self-renewal of CSCs in melanoma by increasing the level of TGF-β expression, which then facilitates the induction of glucosylceramide synthetase through the PKCα/P38/c-Fos signaling pathway (148).

### Therapeutic resistance

#### Drug resistance mechanisms:

The TME contributes to MSC-mediated drug resistance through multiple mechanisms, including the secretion of survival-promoting cytokines (149, 150), EVs released by melanoma cells (151), the induction of autophagy (152), and the activation of pro-survival signaling pathway (13, 153). For instance, growth factors like epidermal growth factor (EGF) and fibroblast growth factor (FGF) activate signaling pathways like the PI3K/AKT and MAPK pathways, promoting CSCs proliferation (154). The hypoxic environment and ECM components can also act as physical barriers, reducing drug penetration to MSCs (155). In addition, CAFs create a resistance niche by interacting closely with CSCs, secreting factors such as IL-6 and IL-8 that promote CSC survival (156).

**Figure 4:**
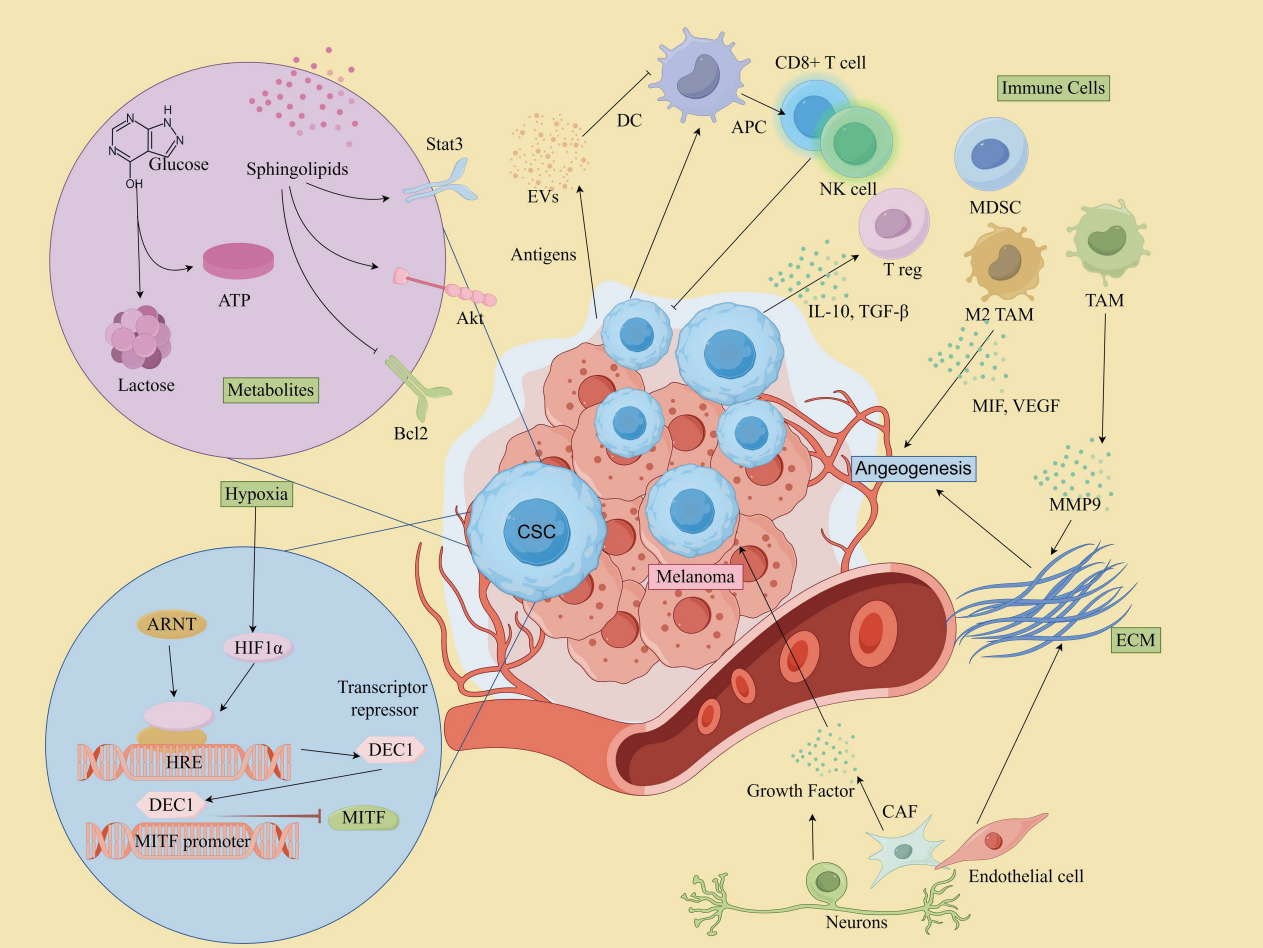
CSCs and microenvironment in melanoma. (By Figdraw).

The intricate interactions between MSCs and various components of the TME contribute to a dynamic environment that fosters melanoma progression and resistance to therapy. These relationships are key to maintaining the supportive niche that allows the tumor to thrive despite treatment efforts. Therefore, a deeper understanding of these complex mechanisms is essential for developing targeted strategies to disrupt this tumor-supportive environment and enhance therapeutic outcomes. A depiction of the TME involving MSCs is provided in **Figure 4**, illustrating the critical elements and interactions within this microenvironment.

## Therapeutic strategies related to MSCs

**Figure 5:**
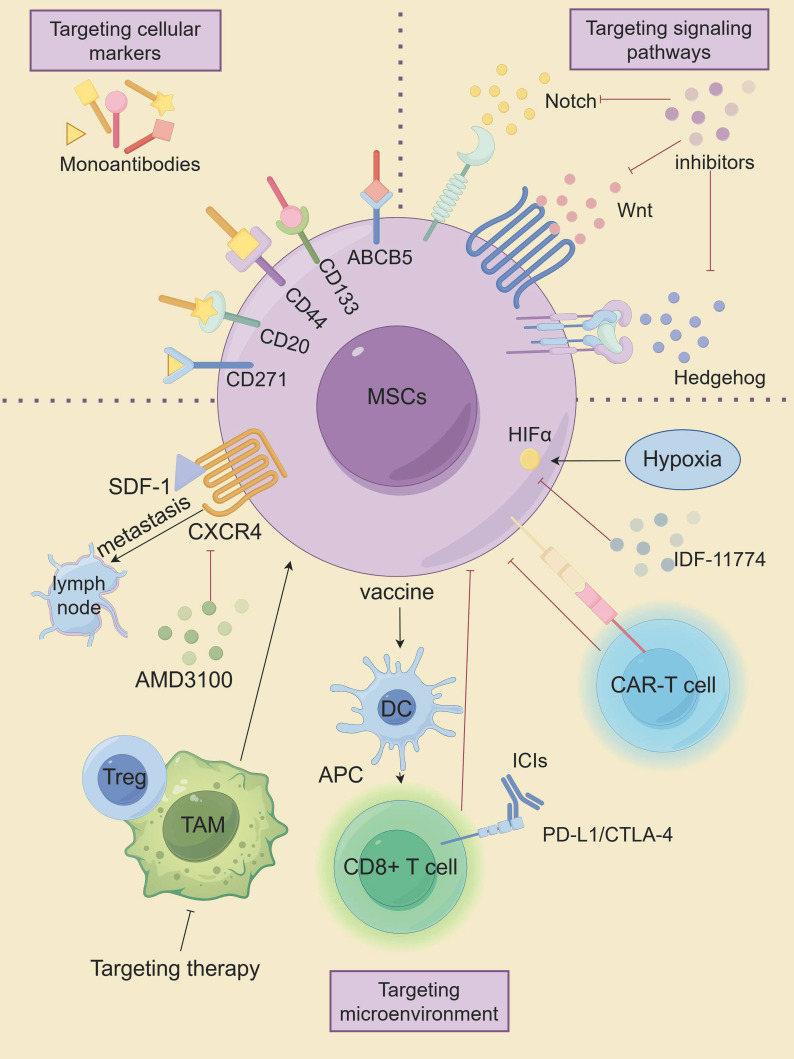
The innovative therapeutic approaches targeting MSCs. (By Figdraw).

Advanced metastatic melanoma is a very challenging cancer because it is resistant to conventional therapies. Conventional anti-cancer therapies focus on eradicating vast populations of tumors that have different properties than the CSC subpopulation. CSCs exhibit remarkable resistance to apoptosis and DNA damage repair mechanisms, enabling evasion of conventional tumor therapies such as radiotherapy and chemotherapy, which are the root cause of tumor recurrence and metastasis. Therefore, targeting MSCs is expected to be a novel approach in the treatment of melanoma, as shown in **Figure 5**, which highlights innovative therapeutic strategies targeting MSCs.

### Targeting cellular markers of MSCs

Cellular markers of MSCs are closely linked to their tumorigenic potential, making them a promising target for therapeutic intervention. Targeting specific stemness markers on MSCs has shown potential in suppressing melanoma progression. For instance, monoclonal antibodies directed against ABCB5 have been demonstrated to induce antibody-dependent cell-mediated cytotoxicity (ADCC) in ABCB5-positive MSCs. Systemic administration of these antibodies has resulted in significant tumor-suppressive effects (157, 158). Similarly, monoclonal antibodies targeting distinct epitopes of the CD133 protein have exhibited cytotoxic effects on melanoma cells, effectively inhibiting tumor growth (159). Suppression of CD133 expression was further associated with reduced metastatic potential and decreased levels of MMP2 and MMP9 (159, 160). CD44, another stemness marker, is expressed across various CSC populations, including melanoma. Shen et al. reported that lipid nanoparticles coated with hyaluronic acid (HA)—a natural ligand for CD44—and loaded with paclitaxel analogs (PTX-loaded HA-SLNs) induced significant apoptosis in CD44-positive B16F10 melanoma cells, both *in vitro* and *in vivo (* 28, 161). As mentioned earlier, CD20 is a well-known cell surface marker of MSCs. Schlaak et al. treated a patient with chemotherapy-refractory metastatic melanoma with the anti-CD20 monoclonal antibody rituximab in combination with low-dose dacarbazine, which showed that the patient achieved complete remission of all but one metastasis that was partially stabilized, that serum malignant black markers S-100 were reduced to normal levels, and that no treatment-related toxicity was observed (29). Moreover, Morita and colleagues developed a humanized anti-CD271 monoclonal antibody (hCD271 mAb) that specifically targeted CD271-positive MSCs. Their *in vivo* studies revealed that this antibody significantly reduced melanoma growth in xenograft models by depleting the CD271-positive cell subpopulation (162). These findings suggest that targeting MSC-specific markers could provide a means to impair tumor growth and reduce recurrence rates in patients.

### Targeting aberrant signaling pathways in MSCs

The maintenance of biological characteristics and self-renewal of MSCs is closely related to multiple signaling pathways (60). Key pathways such as Hh, Notch, and Wnt/β-catenin, which are essential for the formation of melanocyte lineages, have been shown to be strongly associated with the malignant behavior of melanoma cells (163). Inhibition of the Hedgehog pathway, particularly targeting the activator smoothened (SMO) using small interfering RNA (siRNA) and the small molecule inhibitor NVP-LDE-225, has been effective in curbing melanoma growth *in vitro*. Additionally, NVP-LDE-225 not only induced apoptosis *in vitro* but also inhibited tumor growth in xenograft models (164). SMO inhibitors like Vismodegib and Sonidegib have been approved for clinical use; however, their effectiveness has been limited by the development of drug resistance and notable side effects (165). Recent research by Pietrobono et al. led to the development of novel SMO inhibitors based on acylguanidine or acylthiourea scaffolds, which specifically target SMO in melanoma cells, thereby reducing Gli1 expression, inducing DNA damage and apoptosis, and inhibiting the self-renewal of MSCs (166). In the presence of Hh pathway repression, gene expression data also revealed compensatory upregulation of two other developmental pathways, Notch and Wnt (164). The Notch signaling pathway has also been explored as a therapeutic target. Chanh et al. demonstrated that RO4929097, a gamma-secretase inhibitor, reduced the oncogenicity and stem-like properties of melanoma cells *in vitro*, suggesting that Notch inhibition could be a promising strategy for targeting MSCs (67). Moreover, Notch inhibitors have shown potential as radiosensitizers, as their combination with radiation reversed the radiation-resistant phenotype of melanoma *in vitro* and reduced cell migration (167). Additionally, the FDA-approved antipsychotic drug Pimozide has shown efficacy in inhibiting the Wnt/β-catenin signaling pathway, displaying specific cytotoxicity against cancer cells, including melanoma (168). Previous studies have demonstrated that DAPT (a Notch inhibitor), Cyclopamine (an Hh signaling inhibitor), and XAV939 (a Wnt signaling inhibitor) (169), which target CSCs, may represent potentially effective strategies for treating patients with melanoma (22). In addition, Demcizumab (anti-Notch ligand, DLL4 antibody), OMP-52M51 (anti-Notch1 antibody), and OMP-18R5 (anti-Wnt receptor, FZD monoclonal antibody) are expected to be better therapeutic agents in the future against melanoma (21, 22).

While these approaches hold the potential for disrupting the tumorigenic properties of MSCs, they are not without challenges. The Hh, Notch, and Wnt pathways are integral to normal melanocyte development and other physiological functions, which increases the risk of toxicity when employing inhibitors against these pathways. Therefore, the clinical development of these targeted therapies will require a careful approach, emphasizing precise drug delivery systems and guided administration to minimize off-target effects and improve therapeutic selectivity. Future research should focus on optimizing these delivery strategies and validating their safety and efficacy in clinical settings to ensure that the benefits outweigh potential risk.

### Targeting the microenvironment of MSCs

The diverse biological characteristics of MSCs are closely influenced by their surrounding microenvironment, which plays a crucial role in maintaining their stem-like properties and contributing to therapy resistance (170). Studies have shown that modulating this microenvironment can significantly impact the behavior of MSCs. For example, Kim et al. have demonstrated that SDF-1/CXCR4 played an important role in the lymphatic metastatic microenvironment of chemoresistant melanoma cells. Targeting this axis could potentially inhibit the lymphatic metastasis of CD133(+) chemoresistant melanoma cells, offering a promising therapeutic approach (171). Moreover, research indicates that TAMs can interact with CSCs, including MSCs, to promote therapeutic resistance. Mitchem et al. showed that targeting TAM could effectively overcome CSC-mediated therapeutic resistance (172).

In addition to TAMs, other immune cells, such as Treg, are also capable of promoting stemness and treatment resistance in tumor stem cells and can serve as potential targets for the immune microenvironment modulation of CSCs (86). Targeting the hypoxic microenvironment has also shown promise in treating MSCs, with both HIF-1α and HIF-2α emerging as valuable targets (173). Kim et al. found that the HIF-1α inhibitor IDF-11774 inhibited the growth and metastasis of B16F10 melanoma by downregulating HIF-1α expression (174).

Advances in CAR-T cell therapy have introduced new strategies for targeting MSCs. CAR-T cells engineered to recognize CSC surface markers have shown promising results (175). CAR-T cells targeting TYRP1 demonstrated enhanced anti-tumor activity *in vitro* and *in vivo* in both skin and rare melanoma subtypes, with minimal toxicity observed in preclinical models, and Phase I clinical trial is currently in preparation based on these promising outcomes (176).

Recent studies have also indicated that CSC-based vaccines could inhibit the metastasis of primary tumors by inducing humoral and cellular immune responses that lead to the lysis of these targeted CSCs (177). Yin et al. developed a novel Melanoma stem cells (MSCs)-based vaccine that induces CD8+ T cells to specifically target MSCs. The vaccine was found to promote the maturation of DCs, activate CD8+ T cells, inhibit the expression of CTLA-4, PD-1 and Tim-3, and increase the expression of IFN-γ and GzmB in CD8+T cells. The specific targeted killing effect of the vaccine inhibited melanoma growth and metastasis (178). DCs, as the most powerful antigen- presenting cells, have also been used in experimental models and various clinical trials to induce effective antitumor immune responses (25). For example, a novel vaccine targeting CD133(+) CD44(+) MSCs in melanoma, expressing a 6kDa early secretory antigenic target (ESAT-6) and secreting interleukin (IL)-21, significantly suppressed melanoma growth and metastasis in mice, extending survival (179).

ICIs and monoclonal antibodies have reactivated cancer immunotherapy (180, 181). However, while some patients showed partial or even complete responses to immunotherapy, a substantial number did not respond favorably (181). Researchers believed that cancer vaccines may produce tumor-specific T cells, which may be enhanced by ICIs that counteract immunosuppressive mechanisms (182). To investigate this hypothesis, Zheng et al. explored this strategy by combining a MSC-lysate pulsed dendritic cell (MSC-DC) vaccine with PD-L1 and CTLA-4 inhibitors in a mouse model. This dual immune blockade led to significantly greater tumor regression compared to the vaccine alone, suggesting that this combination could enhance immune-mediated anti-tumor responses (182).

## Conclusions and future directions

Melanoma is a kind of skin malignant tumor with high aggressiveness, high metastasis, and poor prognosis. The limited efficacy of conventional treatment modalities, coupled with the emergence of drug resistance and recurrence post-intervention, contributes significantly to the unfavorable therapeutic landscape, thereby constituting a substantial contributor to melanoma-related mortality. The main reason for treatment failure in melanoma patients is the development of tumor heterogeneity, which is due to the formation of genetically distinct subgroups. These subpopulations encompass a minority faction comprising CSCs and a prevailing majority constituting non-tumor stem cells, which collectively compose the tumor mass.

Melanoma contains multiple subpopulations of tumor-initiating cells(TICs), each with different cell surface markers, and promotes melanoma metastasis and drug tolerance by activating a range of signaling pathways. This intricate regulatory network plays a pivotal role in melanoma initiation, progression, and therapeutic resistance. While efforts targeting markers and signaling pathways associated with MSCs have shown promise, challenges persist. The surface markers of MSCs lack sufficient specificity, and the relationship between marker expression, self-renewal capacity, tumorigenic potential, and differentiation remains incompletely understood. Furthermore, comprehensive investigations into the interplay among various markers and signaling pathways are still lacking. Given that these pathways are crucial for both MSCs and normal stem cells, interventions in these signaling pathways may interfere with the function of normal stem cells and cause potential toxicity. High-throughput screening of CSCs offers an objective approach to identify new therapeutic targets specifically for CSCs. Moving forward, it will be essential to carefully distinguish between normal stem cells, CSCs, and non-stem tumor cells to identify specific markers unique to CSCs. This will provide a solid experimental basis for developing targeted therapies. Additionally, strategies like targeted nanoparticles, which can deliver drugs directly to tumor cells, and localized administration methods, which restrict treatment to the tumor site, show great promise in reducing off-target effects and enhancing therapeutic precision. Combination therapy is a major trend in the clinical treatment of tumors, and combining tumor stem cell-targeted therapies with conventional therapies is considered to be a new direction to improve the efficiency of tumor treatment, and provides new opportunities for the treatment of melanoma patients who are resistant to existing therapies and lead to recurrence and metastasis. Moreover, MSCs are closely related to the TME. In addition to adapting to changes in the TME, melanoma can also alter and have an effect on the TME; while the TME can not only affect the self-renewal ability of MSCs, but also induce the transformation of normal cells and non-cancer stem cells to CSCs.

Consequently, only by comprehensively considering the characteristics of all cell subpopulations in tumor tissues and fully grasping the interaction mechanisms of different cell subpopulations in tumors can a more effective and precise tumor immunotherapy protocol be established.

In addition, the current research on MSCs therapy has mostly focused on functional experiments *in vitro*, while relatively few studies were related to their use in humans, and more clinical studies are needed to further support and justify them.
